# Communication on the Sarsaparilla and Sassafras

**Published:** 1833-09

**Authors:** John P. Grant


TRANSACTIONS OF THE MEDICO-BOTANICAL SOCIETY OF LONDON.

*Published under the Direction of the President and Council.*

### SARSAPARILLA AND SASSAFRAS.

### *Communication on the Sarsaparilla and Sassafras.*

By John P. Grant, m.d.

My dear Sir: I have the pleasure to send you herewith specimens of sarsaparilla, which I found growing a few miles from Malacca; and, if cultivated, it would quite supersede the necessity of our importing it from Brazil, at so great an expense as we now do. In small quantities, it can be produced at Malacca, at the rate of a rupee a pound. I have tried it in a number of venereal cases, both primary and secondary, with as much satisfaction from its use as from any I have seen imported from Brazil. The characters of the plant are quite similar in all respects to that of Brazil. It is reared at Malacca by an old Portugueze doctor, who has the sole monopoly of it, and who has successfully reared and used it there for many years. Also a specimen of the Laurus Sassafras, which grows on the top of the hill on this island, and all along the Tennasserrin coast, in great abundance; so much so, that some beams of my house at Tavoy were made of it. It is stronger in flavour, and of a darker yellow, than that imported from America. As it can be supplied for the expense of cutting, we should never go to the expense of importing it from foreign colonies, when we have it in such abundance in our own.

The branch of a tree I send you is the Margosa, which I extensively use in practice. I brought it into notice during the Burmese war, as an application in ulcer. I have noticed its effects at some length in my "Medical Notes," now in the press. I procured the recipe for making the ointment of it from the head priest at Rangoon; it is as follows.

Tender leaves of the Margosa tree, dried and powdered, ʒij.; fresh butter, ℥ij.; wax, ℥ij.; red precipitate, ℥ss. Mix them well, and put the whole into a chatty over a brisk fire for forty minutes, until it assumes a grey appearance. Wash the sore with nitric acid diluted with twenty parts of water, or a strong decoction of the root of the Margosa tree, and apply the dressing, spread on cloth, with a soft compress and rolling bandage, four inches wide, from the toes to the knee, if the ulcer is in the lower part of the leg; and it always has a most wonderful effect in producing healthy granulations.

The bruised leaves are also an excellent application as a fomentation in sprains and bruises, either in man or beast.

The pounded leaves, mixed with lime-juice and salt, are a certain and rapid cure of the ringworm.

I beg also to state, for the information of the Medico-Botanical Society of London, that the Amomum Cardamomum grows in great abundance and perfection in our lately ceded Burmese province on the Tennasserim coast, Tavoy: these can be furnished in seed cleaned from the husk for one shilling per pound.

To Lieut. Mat. Curling Friend, R.N., F.R.S.,
Corresponding Member Medico-Botanical Society of London.

